# Hypoxia Shapes Autophagy in LPS-Activated Dendritic Cells

**DOI:** 10.3389/fimmu.2020.573646

**Published:** 2020-11-30

**Authors:** Sara Monaci, Carlo Aldinucci, Daniela Rossi, Gaia Giuntini, Irene Filippi, Cristina Ulivieri, Giuseppe Marotta, Silvano Sozzani, Fabio Carraro, Antonella Naldini

**Affiliations:** ^1^Cellular and Molecular Physiology Unit, Department of Molecular and Developmental Medicine, University of Siena, Siena, Italy; ^2^Department of Life Sciences, University of Siena, Siena, Italy; ^3^Cellular Therapy Unit and South-East Tuscany Blood Establishment, University Hospital, Siena, Italy; ^4^Laboratory Affiliated to Istituto Pasteur Italia-Fondazione Cenci Bolognetti, Department of Molecular Medicine, Sapienza University of Rome, Rome, Italy; ^5^Department of Medical Biotechnologies, University of Siena, Siena, Italy

**Keywords:** hypoxia, dendritic cell, autophagy, (macroautophagy), hypoxia-inducible factor (HIF)-1α, lipopolysaccharide (LPS)

## Abstract

During their lifespan, dendritic cells (DCs) are exposed to different pO_2_ levels that affect their differentiation and functions. Autophagy is one of the adaptive responses to hypoxia with important implications for cell survival. While the autophagic machinery in DCs was shown to impact signaling of TLRs, its regulation by the MD-2/TLR4 ligand LPS is still unclear. The aim of this study was to evaluate whether LPS can induce autophagy in DCs exposed to either aerobic or hypoxic conditions. Using human monocyte-derived DCs and the combination of immunofluorescence confocal analysis, measure of mitochondrial membrane potential, Western blotting, and RT-qPCR, we showed that the ability of LPS to modulate autophagy was strictly dependent upon pO_2_ levels. Indeed, LPS inhibited autophagy in aerobic conditions whereas the autophagic process was induced in a hypoxic environment. Under hypoxia, LPS treatment caused a significant increase of functional lysosomes, LC3B and Atg protein upregulation, and reduction of SQSTM1/p62 protein levels. This selective regulation was accompanied by activation of signalling pathways and expression of cytokines typically associated with DC survival. Bafilomycin A1 and chloroquine, which are recognized as autophagic inhibitors, confirmed the induction of autophagy by LPS under hypoxia and its impact on DC survival. In conclusion, our results show that autophagy represents one of the mechanisms by which the activation of the MD-2/TLR4 ligand LPS promotes DC survival under hypoxic conditions.

## Introduction

Tissue hypoxia occurs in many physiological and pathological conditions, including lymphoid organs, inflammation, and cancer (1, 2). With regard to lymphoid tissues, oxygen tensions are lower than would be expected from the pO_2_ of inspired air (159 mm Hg or a concentration of 21%, at sea level), of arterial and venous blood, being about 97–100 mmHg and 40 mm Hg, respectively (3). Indeed, the pO_2_ is ∼10 mm Hg in the thymus (4), and the bone marrow and lymph nodes present hypoxic areas, represented by immunological niches (5). Dendritic cells (DCs) are the most effective antigen presenting cells and, based on their differentiation and maturation states, they can be represented as immature and mature (6). Immature DCs circulate through tissues and lymphoid organs, while mature DCs are deputed to initiate the innate and adaptive immune response (7). Thus, DCs, as well as other immune cells, experience different physiological pO_2_ levels ranging from 10 mmHg to 75–100 mmHg (8). When DCs patrol inflamed and cancerous tissues, they are exposed to further lower oxygen tensions, sometimes below 10 mm Hg or less (9), and their functions may be profoundly affected (10). Indeed, the pO_2_ has been found to be important for DC differentiation and especially during final maturation by the MD-2/TLR4 ligand LPS (11, 12). Many of the adaptive responses to hypoxia are mediated by a family of transcription factors known as hypoxia inducible factors (HIFs) (13) and include modulation of glycolytic metabolism, cell survival and migration, pro-angiogenic cytokines, and pro- and anti-apoptotic molecules (14, 15). The HIF dimeric complex is comprised by the constitutively expressed HIF-1β subunit, which associates with one of two hypoxia inducible α subunits, HIF-1α or HIF-2α (16). HIF-1α is expressed ubiquitously and is involved in the inflammatory response in both hypoxic and normoxic conditions (3). Autophagy is one of the adaptive cellular responses to hypoxia. Specific HIF targets in autophagy include BNIP3, a Bcl-2 superfamily member, which modulates cell survival (17). Indeed, cells rely on autophagy to survive diverse cellular insults such as hypoxia, nutrient depletion, accumulation of protein aggregates, damaged mitochondria, or intracellular bacteria (18). Autophagy is a complex self-degradative process that involves several key steps (19).

During macroautophagy (hereafter referred to as autophagy), cytoplasmic material, including organelles, protein aggregates, or bacteria, is sequestered into double membrane–coated autophagosomes. The formation of phagophore is controlled by Beclin-1/VPS34 in response to various types of cellular stress stimuli. Subsequently, the Atg5–Atg12 conjugation is followed by its interaction with Atg16L and multimerization at the phagophore level. LC3B is then processed and inserted into the extending phagophore membrane. The lipidated form of LC3B, LC3B-II, interacts with SQSTM1/p62, a multi-functional adaptor molecule that promotes turnover of poly-ubiquitinated protein aggregates. This is followed by the fusion with endosomes and lysosomes to form autolysosomes where lysosomal degradation can occur (18). An increasing number of recent studies has characterized the involvement of autophagy in DC functions in various physiological and pathological contexts (20). Recently, it has been shown that signaling through TLRs can affect the autophagic process (21). However, while autophagy was shown to impact downstream signaling through some TLRs (TLR4, TLR7, and TLR8), its regulation by TLRs is less clear (20, 22). We have previously shown that hypoxia affected immune cell survival (23, 24). More interestingly, we have previously reported that hypoxia promoted a proapoptotic program in immature DCs (25). The aim of the present study was to investigate whether LPS may activate an autophagic program in hypoxic DCs. We here report that while under normoxia LPS inhibited autophagy, under hypoxia LPS induces increased functional lysosomes, along with modulation of the adapter protein SQSTM1/p62, of LC3B and the protein levels of Atgs, which are all known as markers of autophagy (26–28). All these observations, which were associated with the activation of pro-survival signaling pathways and cytokine expression, were abolished by treatment with two autophagy inhibitors: Bafilomycin (Baf A1) and chloroquine (CQ). The first one is a selective inhibitor of vacuolar-type H ^+^ ATPase (V-ATPase) that blocks the autophagic flux by inhibiting autolysosome acidification and autophagosome–lysosome fusion (29). The second one mainly inhibits autophagy by impairing autophagosome fusion with lysosomes rather than by affecting the acidity and/or degradative activity of this organelle (30). The overall results indicate that the ability of LPS to regulate DC autophagy is tightly related to tissue localization, physio-pathological conditions, and relative local oxygen tensions.

## Materials and Methods

### Reagents

RPMI 1640, fetal bovine serum (FBS), penicillin/streptomycin, and L-Glutamine were purchased from Euroclone, Devon, UK. Fycoll was purchased from Cederlane Labs and Percoll from Amersham Bioscience, Pittsburgh, PA, USA. Recombinant human granulocyte macrophage colony stimulating factor (GM-CSF) and interleukin-13 (IL-13) were purchased from ProSpec TechnoGene, East Brunswick, NJ, USA. All reagents contained <0.125 endotoxin units/ml, as checked by the Limulus Amebocyte Lysate assay (Cambrex, East Rutherford, NJ, USA). LPS from Escherichia coli strain 055:B5 was obtained from Sigma–Aldrich, Milano, Italy. Baf 1A was purchased from VWR Chemicals BDH Milano, Italy, and CQ was obtained by Enzo Life Sciences, Plymouth Meeting, PA, USA.

### Human Monocyte-Derived DC Preparation and Culture Conditions

Human monocyte-derived DCs were generated as previously described (25). Briefly, highly enriched blood monocytes (>95% CD14) were obtained from anonymous buffy coats (through the courtesy of the South-East Tuscany Blood Establishment, AOUS, Siena) by Fycoll and Percoll gradient centrifugations. Monocytes were differentiated into immature DCs (>90% CD1a and < 5% CD14) upon 6 days culture (in RPMI 1640, supplemented with 10% FBS) with 50 ng/ml GM-CSF and 20 ng/ml IL-13, as previously reported (31). Immature DCs were then induced to terminal differentiation with LPS (100 ng/ml) for 24 h and cultured under either normoxia (atmospheric pO_2_ levels: 21% O_2_, 5% CO_2_, and 74% N_2_ corresponding to a pO_2_ ~ 140 mmHg) or hypoxia (2% O_2_, 5% CO_2_, and 94% N_2_, corresponding to a pO_2_ ~ 14 mmHg) by the workstation InVIVO O_2_ 400 (Ruskinn, Pencoed, UK) as previously described (32). In some experiments, cells were treated with Baf A1 or CQ. Briefly, 100 nM Baf A1 or 100 µM CQ were added directly to the culture medium 6 h before the end of treatment (LPS under hypoxia). At the indicated times, cells were harvested for further analysis, as described below.

### Cell Viability and Detection of Mitochondrial Membrane Potential

Cell viability was analyzed by Trypan Blue exclusion assay by Bio-Rad TC20™ automated cell counter (Biorad laboratories, Bio-Rad, Hercules, CA, USA), which provides a total cell count, and it assesses cell viability using a digital image analysis algorithm (33). Evaluation of mitochondrial membrane potential (ΔΨm) was performed by a fluorogenic lipophilic cation (JC-1; Sigma-Aldrich), according to the manufacturer’s protocol as previously described (34). In cells with hyper-polarized mitochondrial membranes, JC-1 spontaneously forms complexes (J-aggregates) emitting red fluorescence. Fluorescence was detected by using microplate reader Fluoroskan Ascent (Thermolabsystem, Helsinki, Finland) protected from light. The ΔΨm was determined by the ratio between the red (~590 nm) and the green (~529 nm) fluorescent emission.

### Immunofluorescence Staining and Confocal Microscope Analysis

DCs were plated on sterile chamber slides (Nunc Lab-Tek) cultured and treated as indicated above. At the indicated time, cells were fixed in cold methanol at −20°C for 10 min and permeabilized with HEPES/Triton for 3 min. Then they were washed with PBS-BSA 0.2% and blocked with 10% goat serum. Cells were incubated with the primary antibody diluted in PBS-BSA 2% anti-HIF-1α (Thermo scientific, Rockford, USA, 1:200 Cat.n° MA-516), LC3B (Cell Signaling Technologies, Danvers, MA, 1:200 Cat.n° 2775S), SQSTM1/p62 (Cell Signaling Technologies, Danvers, MA.1:100 Cat.n°7695), or Atg12 (GeneTex, USA,1:1000 Cat.n° GTX629815) overnight at 4°C in a humidified chamber. The following day, cells were incubated with Cy2 (green) (Jekson Laboratories, 1:5000 Cat.n°711-225-152) or Cy3 (red) (Jekson Laboratories, 1:5000 Cat.n°111-166-045) conjugated secondary antibodies for 1 h at room temperature. Nuclei were visualized by DAPI (Calbiochem, San Diego, CA 1:10000 Cat.n° D9542-1MG). Coverslips were mounted on slides and imaged with LSM-510 META confocal microscope (Carl Zeiss, Oberkochen, Germany). The fluorescence intensity was determined by ImageJ software as the mean pixel density of staining area in each cell. After subtraction of background, the intensity values were shown as arbitrary units relative to control: CTCF (corrected total cell fluorescence) = Integrated Density – (Area of selected cell X Mean fluorescence of background readings).

### Lysotracker Staining

Cells were plated on 8-well coverglass slide (Sarstedt, Germany Cat.n° 94 6190802) and treated with LPS under normoxic or hypoxic conditions. For Baf A1 and CQ treatment, the compounds were added at a concentration of 100 nM and 100 µM, respectively, 6 h before the end of the experiment. After 24 h, cells were labeled by Lyso-ID Green Detection Kit (Enzo Life Sciences, Plymouth Meeting, PA, USA), and nuclear staining was performed by using DAPI. Cells were analyzed by confocal microscope and the fluorescence intensity was determined by ImageJ software, as described above.

### Immunoblotting and Antibodies

DCs were lysed directly in tissue culture plates and processed, as previously described (33). Protein concentration was determined using Micro BCA Protein Assay Reagent kit (Rockford, USA) and equal amounts of total proteins were loaded onto SDS-PAGE gel. After transferring, PVDF membranes were incubated with the specific primary antibodies over night at 4°C: HIF-1α (BD Biosciences, San Jose, CA, 1:200 Cat.n° 610958), Bax (Cell Signaling Technologies, Danvers, MA,1:1000 Cat.n° 2772), Bcl-xl (Cell Signaling Technologies, Danvers, MA,1:1000 Cat.n°2764), LC3B (Cell Signaling Technologies, Danvers, MA,1:1000 Cat.n°2775), Beclin-1 (Cell Signaling Technologies, Danvers, MA,1:1000 Cat.n°3495), Atg3 (Cell Signaling Technologies, Danvers, MA,1:1000 Cat.n°3415), Atg5 (Cell Signaling Technologies, Danvers, MA,1:1000 Cat.8540), Atg7 (Cell Signaling Technologies, Danvers, MA,1:1000 Cat.n°8558), Atg12 (Cell Signaling Technologies, Danvers, MA,1:1000 Cat.n°4180), phNFKB (Cell Signaling Technologies, Danvers, MA,1:1000 Cat.n°3033), phAkt (Cell Signaling Technologies, Danvers, MA,1:1000 Cat.n°4058), php38 MAP Kinase (Thr180/tyr182) (Cell Signaling Technologies, Danvers, MA,1:1000 Cat.n°9211), php44/42 MAP Kinase (Thr202/Tyr204) (Cell Signaling Technologies, Danvers, MA1:1000 Cat.n°9101), PARP (Cell Signaling Technologies, Danvers, MA1:1000 Cat.n°9542), and β-actin (Sigma-Aldrich, 1:50000 Cat.n° A3854). Anti-mouse IgG HRP (Cell Signaling Technologies, Danvers, MA, 1;2000 Cat.n°7076) and anti-rabbit IgG-HRP (Cell Signaling Technologies, Danvers, MA, 1;2000 Cat.n°7074) were used as secondary antibodies (Cell Signaling Technologies, Danvers, MA). Detection of images was performed by ChemiDoc™ MP System (Bio-Rad, Hercules, CA). The intensity of the band was quantified using Image Lab software (Bio-Rad).

### RNA Extraction and RT-qPCR

Total RNA was extracted using EuroGOLD™Trifast reagent (Euroclone, Devon, UK) and cDNA was synthesized using iScript™cDNA Synthesis Kit (Bio-Rad Laboratories). RT-qPCR was performed using iTaq™SYBR Green Supermix (Bio-Rad Laboratories). mRNA levels of BNIP3, VEGF-A, IL-1β, IL-18, TNF-α, IL-6, IL-10, and TGF-β were determined by MiniOPTICON™ System (Bio-Rad Laboratories) and analyzed on an iQ5™ Optical System Software (Bio-Rad Laboratories). Relative quantification was done by using the 2^-ΔΔCT^ method (35) and β-actin as housekeeping gene. Primers were validated as previously described (36).

### Statistical Analysis

The data are presented as the mean ± SEM of at least 3 independent experiments. Statistical analyses were performed with Graph-Pad Prism (San Diego, CA, USA). Analysis of variance (ANOVA) and unpaired two-tailed Student’s t test were used to test for significant numerical differences among the group. Difference of p ≤ 0.05 was considered to be statistically significant (*p ≤ 0.05; **p ≤ 0.01).

## Results

### Hypoxia Affects Autophagy in DCs

**Figure 1:**
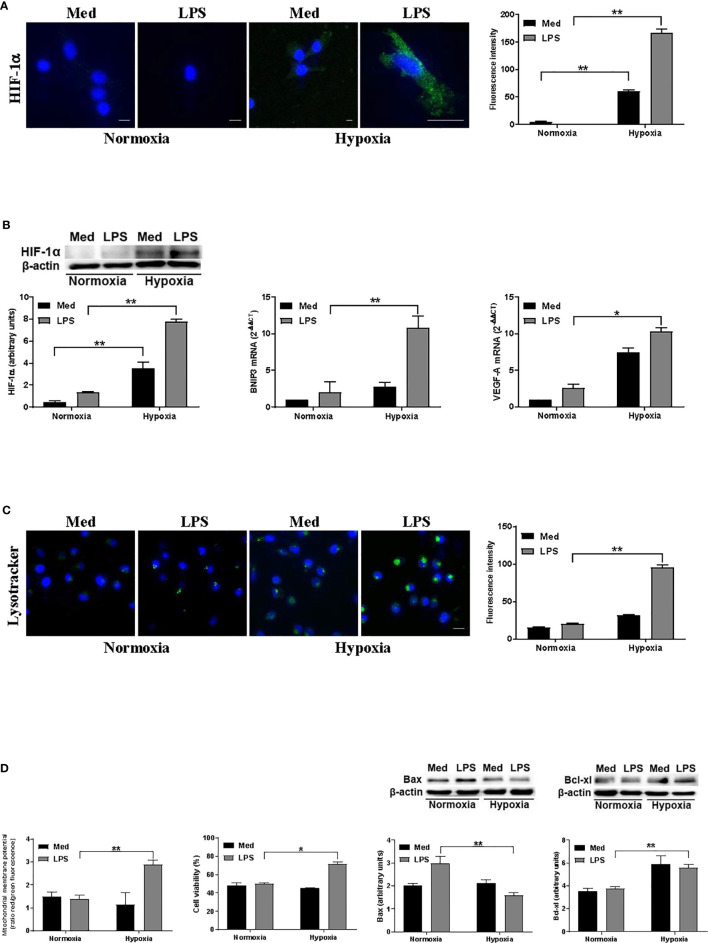
Hypoxia affects autophagy and DC survival **(A)**. HIF-1α protein levels after 24 h exposure to normoxia and hypoxia as determined by confocal microscopy analysis (Scale bar: 8 µm; 15 µm only for LPS in hypoxia) **(B)**. HIF-1α protein levels as determined by Western blotting (blot shown is representative of four independent experiments and β-actin was used as loading control) and RT-qPCR analysis of BNIP3 and VEGF-A mRNA expression (β-actin was used as a housekeeping gene) **(C)**. Detection of acidic/lysosomal compartments by Lysotracker and confocal analysis (Scale bar: 15 µm) **(D)**. Mitochondrial membrane potential analysis by JC-1 dye, DC viability, and Western blot analysis of Bax and Bcl-xl protein levels, under normoxic or hypoxic conditions at 48 h. * and ** indicate statistically significant differences (p ≤ 0.05 and p ≤ 0.01, respectively; n = 4).

In previous reports, we have shown that hypoxia affects DC cell death through the activation of a pro-apoptotic program that was antagonized by LPS (25). Thus, to investigate whether such a protection was associated with autophagy, we exposed human monocyte-derived DCs to a pO_2_ of 140 mmHg (normoxia) or 14 mmHg (hypoxia), either in the presence or not of the MD-2/TLR4 ligand LPS. **Figure 1A** shows that hypoxia significantly enhanced HIF-1α at protein level, especially in the presence of LPS, as observed by confocal microscopy and Western blot analysis. This was paralleled by an increased expression of genes associated with hypoxia, including VEGF-A and BNIP3 (**Figure 1B**). The latter is strictly controlled at transcriptional level by HIF-1α and it is one of the main regulators of autophagy in several cell types upon exposure to hypoxia (17). Since the alterations of lysosome function and of their reformation process is tightly related to autophagy, we then analyzed the amount of acidic/functional lysosomes (37). As shown in **Figure 1C**, confocal analysis revealed a significant increase of acidic vesicles in LPS-treated DCs under hypoxic conditions, as compared to the relative normoxic treatment. We next investigated whether the promotion of autophagy was associated with a modulation of apoptosis. Specifically, we monitored the loss of mitochondrial transmembrane potential (ΔΨ_M_), which is one of the major events associated with apoptosis (38). **Figure 1D** shows that, under hypoxia, LPS treatment resulted in a significantly higher ΔΨ_M_. This was paralleled by an increased number of alive cells, as detected by cell viability assays, the reduction of the pro-apoptotic protein Bax, and enhancement of the antiapoptotic molecule Bcl-xl, thus confirming the protective role of LPS against apoptosis in hypoxic DCs.

### Hypoxia Modulates Autophagy in DCs

**Figure 2:**
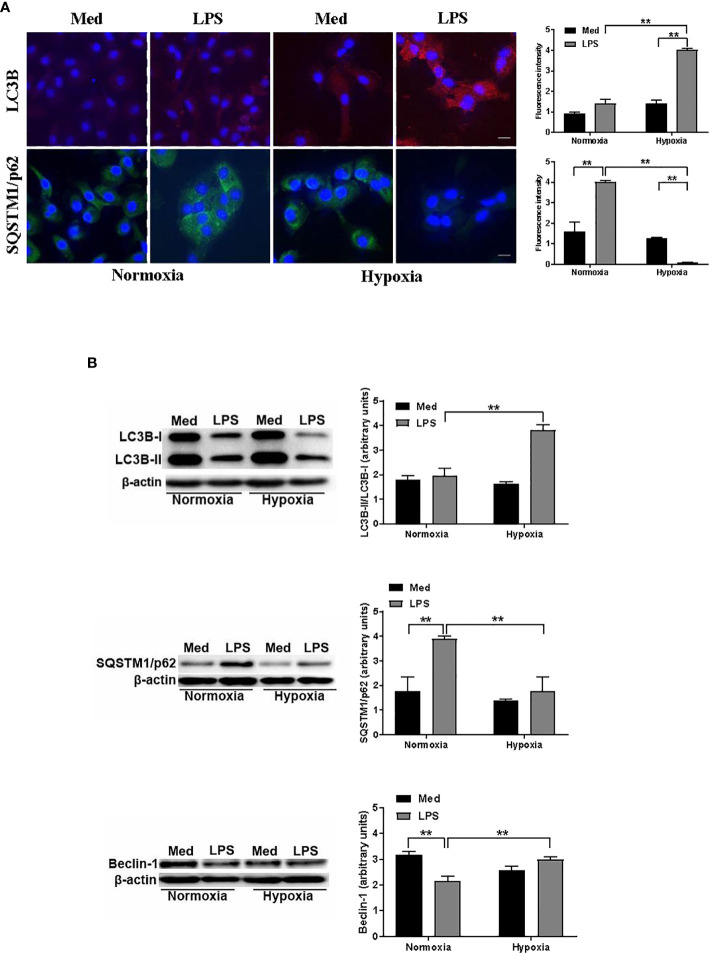
Hypoxia modulates autophagy in DCs **(A)**. LC3B and SQSTM1/p62 protein levels after 48 h exposure to normoxia and hypoxia in DCs stimulated with LPS as determined by confocal microscopy analysis (Scale bar: 15 µm) **(B)**. LC3B-II/LC3B-I, SQSTM1/p62, and Beclin-1 protein levels as determined by western blotting (blot shown is representative of three independent experiments and β-actin was used as loading control). ** indicate statistically significant differences (p ≤ 0.01; n = 4).

To investigate more deeply how hypoxia may affect autophagy in LPS-treated DCs, we next analyzed the protein levels of two key autophagic markers, LC3B and SQSTM1/p62. The localization and aggregation of LC3B, after its conversion to LC3B-II, onto the membranes of autophagosomes is an index of the autophagic flux (39). In **Figure 2A**, confocal immunofluorescent analysis shows that under normoxic conditions the effects of LPS on LC3B-II was not significant. However, LC3B-II was significantly enhanced in LPS-treated DCs under hypoxia. Accordingly, the exposure to hypoxia of DCs in the presence of LPS resulted in a reduced protein level of SQSTM1/p62, indicating a significant increase in autophagy. Indeed, after delivering the autophagic substrates to autophagosomes, SQSTM1/p62 is degraded and its protein level is decreased when autophagy is induced (40). In contrast, LPS treatment under normoxia resulted in accumulation of SQSTM1/p62, suggesting that the induction of the autophagic process occurred only under hypoxic conditions. To corroborate the hypothesis that hypoxia induces autophagy in LPS-treated DCs we next analyzed LC3B-II/LC3B-I ratio by Western blot. **Figure 2B** shows that this ratio was significantly increased in hypoxic LPS-treated DCs, as compared with normoxia. Accordingly, with the immunofluorescent confocal analysis, the protein level of SQSTM1/p62 was significantly enhanced upon LPS treatment under normoxia, while it was reduced under hypoxia, indicating a pro-autophagic process only in the latter condition. The fact that LPS-treated DCs were more prone to autophagy under hypoxia was further confirmed by a significant increase of another marker of autophagy, Beclin-1, which is required for the autophagic flux induction (41).

### Hypoxia Modulates the Atg Protein Levels in DCs

**Figure 3:**
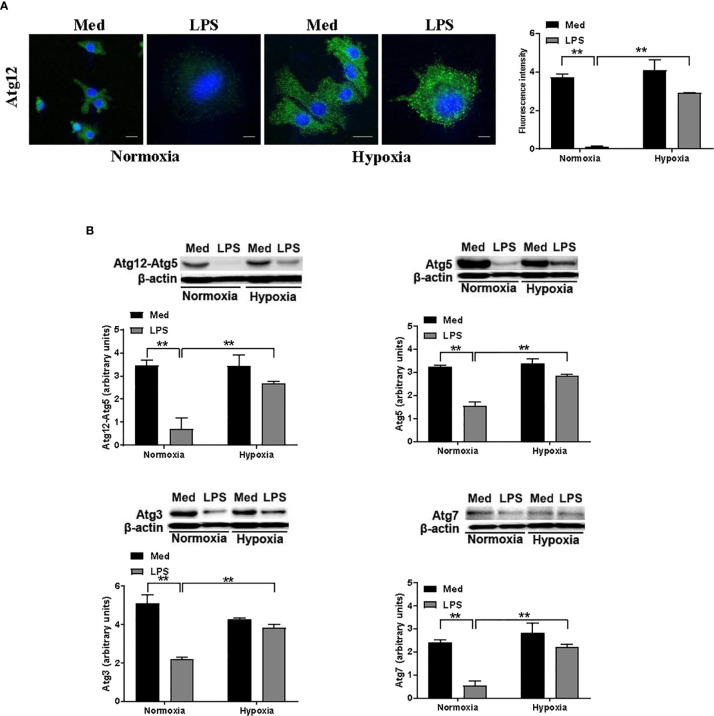
Hypoxia modifies the levels of Atg proteins in DCs **(A)**. Atg12 protein levels after 24 h exposure to normoxia and hypoxia in the presence of LPS, as determined by confocal microscopy analysis (Scale bar: 15 µm for medium, normoxia, and hypoxia, and 4 µm for LPS, in normoxia and hypoxia) **(B)**. Atg12-Atg5, Atg5, Atg3, and Atg7 protein levels as determined by western blot analysis (β-actin was used as loading control). The blots are representative of three independent experiments. ** indicate statistically significant differences (p ≤ 0.01; n = 3).

Since several reports indicate the involvement of Atgs in the functional aspects of DC maturation, we next analyzed the effect of hypoxia on the level of several Atg proteins in DCs treated with LPS. We first analyzed the protein level of Atg12 by confocal immunofluorescent analysis. Atg12, along with Atg5, upon binding to Atg16, is essential for autophagosome elongation and it is downstream of Beclin-1 (28). As shown in **Figure 3A**, Atg12 was apparently reduced upon LPS treatment under normoxic conditions. However, when DCs were exposed to hypoxia, LPS treatment resulted in a significant increase of Atg12 protein level, when compared with normoxic LPS-treated DCs. These results were confirmed by Western blot analysis (**Figure 3B**). Indeed, antibodies against Atg12-Atg5 complex and to Atg5 alone revealed a significant reduction in LPS-treated DCs under normoxia, while under hypoxia LPS treatment resulted in a significant increase in the protein levels of both Atgs. Similar results were obtained also for other Atg proteins, including Atg3 and Atg7, which are crucial for autophagosome formation (42). Indeed, under hypoxia, LPS treatment resulted in a significant increase in the protein levels of both Atgs, as compared with the relative normoxic controls. Even in these cases, when DCs were treated with LPS under normoxia, we observed a significant decrease in the Atgs that we had analyzed. Of note, in all cases Atg levels of LPS-treated cells were lower than in untreated cells. Indeed, we cannot exclude that, since autophagy is a degradative process, fewer levels of Atgs in hypoxic LPS-treated cells may be reduced by the turnover that is associated with autophagy.

### Hypoxia Affects the Expression of Several Signaling Molecules and Cytokines Associated With DC Autophagy, Cell Survival, and Activation

**Figure 4:**
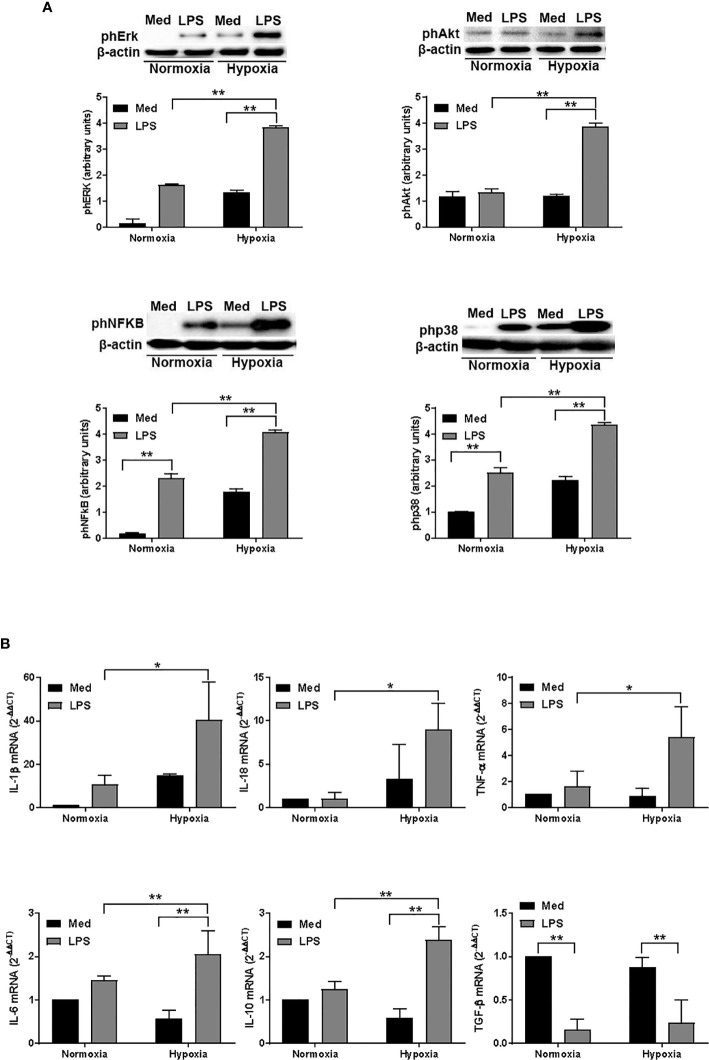
Hypoxia affects signalling pathways and and cytokine expression in DCs **(A)**. Erk, Akt, NFκB, and p38 phosphorylation in DCs after a 24-hour exposure to normoxia and hypoxia with or without LPS, as determined by western blotting (the blots are representative of three independent experiments and β-actin was used as loading control) and **(B)** RT-qPCR analysis of IL-1β, IL-18, TNF-α, IL-6, IL-10, and TGF-β mRNA expression (β-actin was used as housekeeping gene) at the end of 24 h treatment. * and ** indicate statistically significant differences (p ≤ 0.05 and p ≤ 0.01, respectively; n = 4).

To further analyze the impact of hypoxia on LPS-treated DCs, we next analyzed the activation of several signaling pathways associated with DC survival and, more recently, with autophagy (43). As shown in **Figure 4A**, LPS treatment resulted in an increased phosphorylation of Erk in normoxic conditions. However, when DCs were treated with LPS under hypoxia, we observed a significant enhancement of Erk phosphorylation as compared with LPS-treated DCs in aerobic conditions. We observed a similar pattern also for Akt that, along with Erk, is essential to inhibit DC apoptosis and to promote DC survival (44). In addition, LPS treatment under hypoxia resulted in an increased phosphorylation of NFkB and p38. Both pathways are involved in DC maturation and activation, including the expression of several cytokines, which are released by DCs (45, 46). Indeed, LPS-treated DCs expressed significantly higher amounts of IL-1β, IL-18, TNF-α, IL-6, IL-10, and TGF-β mRNA, as measured by RT-qPCR, in both normoxic and hypoxic conditions (**Figure 4B**). However, when DCs were treated with LPS under hypoxic conditions, the expression of cytokine mRNA was significantly higher as compared with normoxia. Of interest, the pattern of expression was similar for all the cytokines that were analyzed, except for TGF-β. Indeed, LPS treatment resulted in a significantly lower expression of TGF-β in both normoxic and hypoxic treatment. This observation, however, was in line with other previous reports (47). The overall results indicate that hypoxia positively regulates DC responses that are associated with their survival, maturation, and functional activation.

### Autophagy Is Involved for LPS-Treated DC Survival

**Figure 5:**
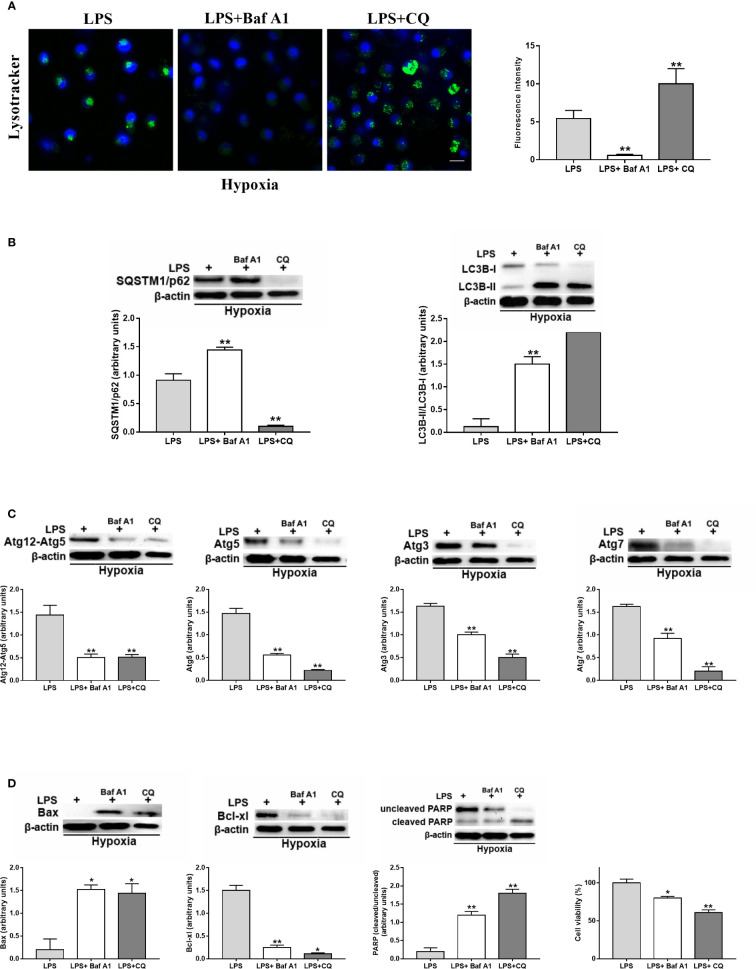
Autophagy is involved in the survival of activated DCs **(A)**. Detection of acidic/lysosomal compartments by Lysotracker and confocal analysis (Scale bar: 15 µm) in DC stimulated with LPS under hypoxia for 24 h with Baf A1 or CQ **(B)**. SQSTM1/p62 and LC3B-II/LC3B-I **(C)**, Atg12, Atg5, Atg3, and Atg7 and **(D)** Bax, Bcl-xl, PARP protein levels as determined by western blotting (blot is representative of three independent experiments and β-actin was used as loading control) and cell viability. * and ** indicate statistically significant differences (p ≤ 0.05 and p ≤ 0.01, respectively; n = 4).

Due to the above observations, we decided to further investigate the potential mechanism by which hypoxia may affect LPS-treated DCs in terms of autophagy. To this end, we evaluated the effects of Baf A1 and CQ by confocal microscopy analysis using the pH-sensitive lysosomal dye LysoTracker in hypoxic LPS-treated DCs. As expected from an inhibitor of the vacuolar proton pump (29), Baf A1 treatment decreased the acidity of lysosomes as it led to a rapid decrease of fluorescence (**Figure 5A**). CQ, in contrast, but in agreement with previous reports, did not decrease LysoTracker-positive structures, which tended to be much larger after CQ treatment compared to control or Baf A1 treatment (30).

Autophagy inhibition by Baf A1 resulted in a higher protein level of SQSTM1/p62 and of LC3B-II/LC3B-I (**Figure 5B**). However, and in agreement with previous reports, the increased amounts of LC3B-II can correlate with either an induction of autophagy or a block at the late steps of this pathway, i.e., autophagosome fusion with lysosomes and/or lysosomal degradation (30). Similarly, CQ treatment resulted in a significant enhancement of LC3B-II/LC3B-I ratio. However, CQ reduced the protein level of SQSTM1/p62, probably due to the fact that CQ does not substantially decrease lysosomal activity (30).

To further test whether Baf A1 and CQ affected the autophagic process in hypoxic LPS-activated DCs, we evaluated the protein levels of several Atgs, which are involved in different steps of the autophagic process (20). **Figure 5C** clearly shows that both Baf A1 and CQ treatments reduced the protein levels of Atg12, Atg5, Atg7, and Atg3.

Finally, as shown in **Figure 5D**, Baf A1 and CQ enhanced the protein level of the pro-apoptotic protein Bax and reduced that of Bcl-xl, which is an anti-apoptotic and pro-survival protein (48). This was associated with a significant increase of PARP cleavage, which is another marker of apoptosis (49). More importantly, the inhibition of autophagy by Baf A1 and CQ resulted in a significant decrease of alive cells.

Thus, the overall results indicate that hypoxia enhanced autophagy in DCs activated by LPS, leading to the promotion of DC survival and activation.

## Discussion

In this paper we described for the first time how hypoxia may affect DC autophagy, with particular regard to DC final maturation induced by the MD-2/TLR4 ligand LPS (50).

We and others have previously shown that hypoxia significantly affects T cell and DC functions, with important physiological and pathological implications in the immune response (32, 51–53). Concerning DCs, we have previously shown that hypoxia promotes a pro-apoptotic program in immature DCs (25). However, in the same study, when hypoxic DCs were maturated with LPS, we did not observe an increase in cell death, while HIF-1α accumulation and BNIP3 expression were still significantly upregulated. The purpose of the present manuscript was to investigate whether LPS-treated DCs may undergo a pro-autophagic program. Accordingly, we here report that human derived DCs, treated with LPS, were more susceptible to autophagy under hypoxia (pO_2_ = 14 mmHg, 2%O_2_) as compared with the aerobic condition (pO_2_ = 140 mmHg, 21%O_2_). We should underline that the pO_2_, which was employed in our study, is similar to the microenvironmental pO_2_ present in lymphoid tissues (54), in inflammation (5), and in solid tumors (55). Such an effect was evident with regard to the number of acidic/functional lysosomes and to the protein levels of molecules associated with autophagy (18). Previous reports indicate that hypoxia promotes autophagy resulting in prolonged cell survival (56, 57). Indeed, the expression of BNIP3, which is transcriptionally regulated by HIF, is tightly related to autophagy (17). Accordingly, we here report that hypoxia enhances BNIP3 mRNA expression along with the protein level of Beclin-1, which is an important marker of the early autophagic program (41). Autophagy is a very complex process that has been associated with DC functions (20). While it is widely accepted that autophagy activates TLR4 downstream signaling, the effect of LPS activation on autophagy is still a matter of debate. Previous studies, which were all conducted under aerobic conditions, reported that TLRs stimulation either promoted (21) or reduced the autophagy flux, in particular, upon the stimulation of primary DCs by LPS (58). Our results are in line with the latter report, since we observed that under normoxic conditions the protein level of SQSTM1/p62 was significantly enhanced by LPS treatment. Furthermore, we did not observe a significant increase of LC3B-II, a phenomenon normally expected during the autophagic process. However, when LPS-treated DCs were cultured under hypoxic conditions, we observed a significant reduction of SQSTM1/p62 protein level, paralleled by an increase of LC3B-II protein level. Thus, the induction of the autophagic process in DCs is strictly related to their maturation state and to the microenvironment in which they localize. The fact that the differentiation and maturation stages are critical for DC autophagy was clearly suggested by several reports (59). Proteins implicated in the elongation and formation of autophagosomes are differently regulated during DC maturation steps (20). The elongation of the phagophore, along with autophagosome formation, is controlled by a series of ubiquitin-like conjugation reactions catalyzed by the E1-like enzyme Atg7 and E2-like enzyme Atg3 (28). Atg7 mediates the binding of Atg12 and Atg5. Of interest, Atg7, Atg5, and Atg3 are critical in DC autophagy and functional activities (22, 42). We here show that the protein levels of these and other Atgs, which were clearly inhibited by LPS under normoxic conditions, were significantly increased under hypoxia. DC survival and activation are commonly associated with several signaling pathways and molecules known to be involved in autophagy (25, 44, 60). Accordingly with the hypothesis that autophagy promotes cell survival and activation in LPS-treated DCs, we here show that hypoxia significantly upregulates phosphorylation of Akt, Erk, p38, and NFkB. While the first two molecules are part of signaling pathways associated with DC autophagy and survival, p38 and NFkB were also associated with DC activation, still in an autophagic context (46, 61). In addition, DC activation by LPS resulted in the expression of several cytokines, with some of them being considered pro-survival factors (62). It should be underlined that the expressions of some of these cytokines, such as IL-1β, IL-18, and TNF-α, are known to be regulated by autophagy (63). By showing the upregulation of these cytokines, this study further supports the hypothesis that hypoxia promotes survival and activation of TLR-activated DCs. However, we cannot exclude the possibility that changes in LPS-induced cytokine mRNA expression seen under hypoxic conditions may be linked to other pO_2_-dependent cellular changes rather than to the observed effects on cellular autophagosomal machinery.

The concept that autophagy is induced by hypoxia was corroborated by two of the most commonly used inhibitors to study autophagy, Baf A1 and CQ (30, 64). Previous reports have shown that Baf A1 treatment severely affected autophagy in several cell types, including bone marrow–derived DCs (65). Accordingly, in our study Baf A1 inhibited the autophagic process in hypoxic LPS-treated DCs by reducing the number of functional lysosomes, upregulating SQSTM1/p62, and downregulating Atg protein levels. In contrast, Baf A1 enhanced the protein level of LC3B-II. This result was, however, in agreement with other studies, reporting an inhibition of autophagy even in the presence of enhanced LC3B-II (66).

Of interest, enhanced LC3B-II levels were also observed upon CQ treatment. It must be pointed out that increased LC3B-II levels can be associated with either enhanced autophagosome synthesis or reduced autophagosome turnover, probably due to delayed trafficking to the lysosomes, reduced fusion between compartments, or impaired lysosomal proteolytic activity (66). This also justifies the results obtained for functional lysosomes, where Lysotracker positive structures tended to be much larger after CQ treatment compared to control or Baf A1 treatment.

Furthermore, CQ treatment resulted also in SQSTM1/p62 reduction. This is in agreement with previous reports showing that the degradative capacity of the cells still remains intact especially upon exposure to CQ, and the lysosomes retain their capacity to degrade delivered material (67).

Keeping in line with the fact that Baf A1 and CQ inhibit autophagy, we observed that both compounds reduced the protein levels of all the Atgs that were analyzed. Finally, and in agreement with several studies showing that autophagy may promote a pro-survival program, Baf A1 and CQ treatments resulted in the modulation of pro- and anti-apoptotic Bcl-2 family proteins (68), in the increased cleavage of PARP, and, more importantly, in the reduction of alive cell numbers. However, further studies are required to better assess which autophagic marker may be involved in the observed effects by using either other inhibitors or specific siRNAs.

The apparent absence of CD14 in the mature DCs exposed to LPS and the critical role of CD14 in efficient delivery of activating LPS to MD-2/TLR4 leaves open the possibility of an alternative mechanism of LPS-induced DC responses that may be not TLR4-dependent. However, previous experimental evidence documents that soluble CD14 from plasma/serum contributes to LPS/TLR4 signalling in CD14-negative cells (69, 70). Still in line with the possibility of an alternative mechanism of LPS-induced DC responses in hypoxia, we should highlight that LPS enhances PI3K/Akt activation in hypoxic DCs and that its abrogation results in an enhanced DC cell death (25). Of interest, previous reports indicate that LPS‐induced phosphorylation of Akt was TLR4‐dependent (71). Thus, future studies are needed to further understand the possible involvement of PI3K/Akt for regulating DC autophagy under hypoxia.

In conclusion, our data indicate that under hypoxic conditions, LPS activation of DCs leads to a pro-autophagic program. Autophagy is crucial for DC orchestration of the immune response and hypoxia is a common feature in pathological conditions, such as inflammation, tumor microenvironment, and within the microenvironment of lymphoid tissues. Thus, this study contributes to the understanding on how DCs adapt to changes of pO2, typically associated with different immune responses, and provides the ground for new future therapeutic regulation of DC functions.

## Data Availability Statement

The raw data supporting the conclusions of this article will be made available by the authors, without undue reservation.

## Ethics Statement

The studies involving human participants were reviewed and approved by Ethical Committee of Azienda Ospedaliera Universitaria Senese (AOUS) and University of Siena. The patients/participants provided their written informed consent to participate in this study.

## Author Contributions

AN, FC, and SM designed the research. SM, CA, GG, IF, CU, and GM performed the experiments. SM, DR, FC, and AN analyzed the data. SM and FC produced the figures. SM, SS, and AN wrote the manuscript. All authors contributed to the article and approved the submitted version.

## Funding

MIUR: PRIN 2017NTK4HY_002 to AN and PRIN 20177J4E75 and AIRC: IG 2017 - ID. 20776 to SS.

## Conflict of Interest

The authors declare that the research was conducted in the absence of any commercial or financial relationships that could be construed as a potential conflict of interest.
